# *Bacillus megaterium* supplementation improves growth performance, immunity, antioxidant capacity, and gut microbiota in cold-stressed neonatal calves

**DOI:** 10.3389/fmicb.2026.1748969

**Published:** 2026-04-02

**Authors:** Mengjian Liu, Zixuan Ye, Xucheng Mo, Yakun Wang, Siyu Li, Yaoli Fu, Yujie Niu

**Affiliations:** 1 College of Animal Science, Xinjiang Agricultural University, Urumqi, Xinjiang, China; 2 Xinjiang Changji National Agricultural High Tech Industry Demonstration Zone, Changji, Xinjiang, China; 3 Animal Nutrition and Feed Science, College of Animal Science and Technology, Shihezi University, Shihezi, China

**Keywords:** antioxidant capacity, *Bacillus megaterium*, diarrhea, immune function, microbiota structure, neonatal calves

## Abstract

**Introduction:**

Neonatal calves exhibit immature digestive and immune systems, rendering them susceptible to environmental stressors such as cold temperatures, which exacerbate gastrointestinal dysfunction and diarrhea incidence. Antibiotic use for mitigation poses risks, including microbiota disruption and resistance development, necessitating safe probiotic alternatives.

**Methods:**

This study evaluated the effects of *Bacillus megaterium* supplementation on growth performance, diarrhea occurrence, serum biochemical, immune, and antioxidant parameters, and rectal microbiota composition in neonatal calves under Xinjiang’s cold climate. Fifty crossbred calves were randomly assigned to five groups (*n* = 10): basal diet (Group I), basal plus 50 mg/day gentamicin (Group II), or basal plus 250, 500, or 1,000 mg/day *B. megaterium* (Groups III–V). Supplementation occurred via milk over 28 days, with assessments of growth performance, fecal scores, serum indices, and rectal microbiota.

**Results and discussion:**

The 500 mg/day *B. megaterium* treatment (Group IV) significantly increased average daily gain (ADG) and reduced feed-to-gain ratio (F/G) and diarrhea frequency compared to control (*p* < 0.05). Serum IgG increased, whereas pro-inflammatory cytokines (IL-1β, TNF-α, IFN-γ) decreased in the probiotic group compared with controls (*p* < 0.05). Antioxidant capacity improved significantly, with GSH-Px and CAT elevated and MDA reduced (*p* < 0.05). Rectal microbiota Shannon index was significantly higher in Group IV compared to the Group II (median: 2.7 vs. 3.8; *p* < 0.05). The relative abundance of Firmicutes increased, and beneficial genera (*Lactobacillus*, *Faecalibacterium*, *Ruminococcaceae_UCG-014*) were enriched, whereas *Escherichia–Shigella* decreased in Group IV (*p* < 0.05). Beneficial taxa were positively associated with immune and antioxidant markers and negatively associated with pro-inflammatory cytokines. Overall, these findings suggest that *B. megaterium* is a promising antibiotic alternative for promoting calf health, productivity, and beneficial gut microbiota under cold stress, with implications for more sustainable ruminant production systems.

## Introduction

In modern cattle production systems, ensuring the health and efficient rearing of neonatal calves is a critical prerequisite for long-term herd productivity and profitability. Neonatal calves represent a critical developmental stage and are born with immature digestive and immune systems (Meale et al., 2017; Du et al., 2023). The structure and function of immune organs in neonatal calves differ significantly from those in adult cattle, characterized by fewer immune cells and reduced cellular functionality (Cangiano et al., 2024). Moreover, the integrity of the intestinal barrier is compromised due to loose tight junctions between intestinal epithelial cells, making calves highly susceptible to environmental stressors such as temperature fluctuations (Yu et al., 2024; Zhang L. et al., 2025; Zhang Z. et al., 2025). Xinjiang, China, provides extensive grasslands suitable for herbivore production. However, its harsh continental climate, characterized by long, cold winters, frequent strong winds, and pronounced diurnal and seasonal temperature fluctuations, imposes considerable stress on young animals (Guan et al., 2022). Beyond general stress, cold exposure can directly compromise the intestinal epithelial barrier by disrupting tight junctions and promoting mucosal inflammation, and it can also remodel gut microbial metabolism in ruminants (e.g., propionate and butyrate pathways), which may increase susceptibility to enteric dysfunction and diarrhea in neonatal calves (Guo et al., 2022; Cheng et al., 2025). These environmental constraints render neonatal calves vulnerable to gastrointestinal dysfunction, immunosuppression, and diarrhea (Chen et al., 2022; Yu et al., 2024). Neonatal diarrhea impairs growth performance by reducing average daily gain (ADG) and feed efficiency, increases veterinary and management costs, and disrupts intestinal development, with adverse consequences for long-term health and production efficiency (Chen et al., 2022; Du et al., 2023). Antibiotics are still widely used to control calf diarrhea, but their application is associated with disruption of the gut microbiota, impaired intestinal and immune development, the emergence of antimicrobial resistance and concerns about drug residues. Therefore, there is an urgent need to develop safe and effective alternatives to in-feed and therapeutic antibiotics in calf rearing (Du et al., 2023; Zhang L. et al., 2025).

*Bacillus megaterium* (*B. megaterium*) is a rod-shaped, gram-positive, strictly aerobic, and motile spore-forming bacterium (Etesami et al., 2023). It produces a wide range of extracellular enzymes that enhance nutrient digestion and absorption (Chen et al., 2018; Elisashvili et al., 2018). Certain strains produce bacteriocins and extracellular polysaccharides, and cell wall glycolipids and glycoproteins have been suggested to influence intestinal development (Chen et al., 2018). In addition, *B. megaterium* can activate immune cells, enhance host immune responses, reduce inflammation, and secrete antimicrobial substances that inhibit pathogens, thereby modulating gut microbiota composition (Avdiyuk and Varbanets, 2023). Evidence from young monogastric models provides supportive but indirect indications of these functions *in vivo*: dietary supplementation with *B. megaterium* has been reported to improve growth performance, optimize gut microbiota composition, and enhance intestinal barrier function in piglets (Bakun et al., 2021), and to be associated with improved immune status and a lower incidence of enteric disorders (including diarrhea) in broiler chickens (Luise et al., 2022). However, because gastrointestinal physiology and microbial ecology differ substantially between monogastrics and ruminants, the efficacy, optimal dosage, and underlying mechanisms of *B. megaterium* in neonatal calves remain largely unclear, particularly under environmental stress conditions.

Therefore, the present study aimed to evaluate the effects of different dietary doses of *B. megaterium* on growth performance, diarrhea incidence, serum biochemical, immune, and antioxidant indices, and rectal microbial community in neonatal calves raised under cold stress conditions in Xinjiang. By integrating growth, health, and microbiota, this research seeks to provide mechanistic insights into how *B. megaterium* modulates the gut–serum axis in neonatal calves and to generate scientific evidence and practical guidance for sustainable and efficient calf rearing in cold regions.

## Materials and methods

### Materials preparation

*B. megaterium* (R&D center of COFCO Grain and Oil Industry Co., Ltd., Changji, China, ccj-bac-meg1801) was cultured in liquid enrichment medium at 37 °C for 48 h with agitation, then precipitated at 4 °C for 24 h. The precipitate was centrifuged at 4,000 rpm for 3 min, and the supernatant was discarded. The pellet was mixed with bacterial suspension, Tween-80, skimmed milk powder, and wheat bran (1:1:2:1, w/w) and lyophilized at −80 °C for 72 h to obtain freeze-dried powder. The freeze-dried powder was confirmed to be primarily in the spore form (>95% spores via microscopic count), and spore counts remained stable (±5%) when stored at 4 °C for 30 days. Prior to the trial, the viability of the powder after reconstitution in warm milk (42 °C) was verified, with a viability loss of <10% over a 30 min period. The final product had a survival rate of 88.47%, yielding a concentration of 2.6 × 10^10^ CFU/g. During the trial, the actual CFU delivered per calf was verified weekly by plating serial dilutions of the milk-probiotic mixture immediately after preparation. Gentamicin was obtained from Ruicheng Lvman Biopharmaceutical Co., Ltd.

The experiment was conducted from November to December 2024, when the average ambient temperature was −13.5 °C and wind speeds of beaufort scale 4–8 occurred 4–6 days per week. The trial was performed at a cooperative calf facility in Emin County, Tacheng, Xinjiang (46°65′N, 83°25′E). The region has a continental temperate climate with short hot summers, rapid cooling in autumn, and long cold dry winters. Average winter temperatures range from −6 °C to −7 °C, with extreme lows reaching −30 °C and heavy snowfall. In spring, the mean temperature is about 7 °C, with large diurnal variation and frequent strong winds.

### Animals and diets

This study employed a single-factor randomized experimental design. Fifty healthy neonatal crossbred calves (Simmental × Xinjiang Brown), blocked by birth weight (40.8 ± 2.6 kg) and birth date, were randomly assigned using a number generator in Microsoft Excel to five groups (*n* = 10 per group; one calf per replicate). Housing, feeding, and immunization followed the cooperative’s standard protocols. Calves were individually housed in separate calf hutches (1.50 × 2.50 m; 3.75 m^2^) within a calf shed, each bedded with clean wheat straw. The temperature in the pen is 6 °C, with a humidity of 62%. Calves received 4 L of warm colostrum within 1 h after birth. From birth to 7 days, calves were fed 6 L/day of milk at 40 °C. From 8 to 28 days, calves received 8 L/day of milk divided into three meals (08:30, 14:30, and 20:30). *B. megaterium* powder was mixed into the milk immediately before feeding. Starter was offered from 7 days of age, and alfalfa was available ad libitum.

*B. megaterium* was provided as freeze-dried powder (2.6 × 10^10^ CFU/g) and mixed with milk warmed to 42 °C. Group I received only the basal diet; Group II received the basal diet plus 50 mg/day gentamicin per calf, a dose chosen based on long-term use at the cooperative. Groups III, IV, and V received 250, 500, and 1,000 mg/day of *B. megaterium* per calf, respectively. The experimental period was 28 days (from 1 to 28 days of age). The diet for neonatal calves consisted of milk, starter feed, and alfalfa. The composition of the starter feed is presented in Table 1. Nutritional levels of each diet are detailed in Table 2.

**Table 1:** Composition of starter feed.

Items	Content (%)
Soybean meal^a^	25
Expended soy	13
Whey powder	5
Corn	25
Expended corn	17.9
Wheat bran	10
CaHPO_4_	0.8
NaCl	0.5
Limestone	1.8
Premix^b^	1
Total	100

a Soybean meal: 89.1% dry matter and 42.6% crude protein.

b The premix provided the per kg of diet as follows: VA 15,000 IU, VD 5,000 IU, VE 50 mg, Fe 90 mg, Mn 60 mg, Cu 12.5 mg, Zn 100 mg, I 2.0 mg, Co 0.5 mg, Se 0.3 mg.

**Table 2:** Milk, starter feed and alfalfa nutrition.

Items	Milk	Starter	Alfalfa
Dry matter (%)	12.63	91.27	94.63
Crude protein (%)	3.19	18.02	14.67
Ether extract (%)	3.92	4.19	1.53
Crude ash (%)	0.69	7.57	9.35
Calcium (%)	0.12	1.09	1.47
Phosphorus (%)	0.09	0.58	0.31
Total energy (MJ/kg)	2.72	17.65	17.98

The nutritional levels were measured.

### Sampling

The amounts of feed offered and residual feed were recorded daily throughout the experimental period. Dry matter intake (DMI) was calculated for days 1–14, 15–28, and 1–28. Calves were weighed on days 1, 14, and 28 before the morning feeding. Average daily gain (ADG) was then calculated for days 1–14 days, 15–28 days, and the overall period (1–28 days). The feed-to-gain ratio (F/G) was calculated as DMI/ADG.

On 14 and 28 days, blood samples were collected from the jugular vein of neonatal calves using sterile vacuum tubes containing 0.1 mL of 0.04% EDTA anticoagulant before feeding. Samples were centrifuged at 3,000 r/min for 15 min at 4 °C. The serum was then separated and stored at −80 °C for subsequent analyses of physiological, biochemical, immune, and antioxidant indices.

One day before the end of the trial, fresh rectal fecal samples (~20 g) were collected using sterile long-arm gloves. Samples were immediately transferred into cryotubes with protective solution and snap-frozen in liquid nitrogen for microbial analysis.

### Fecal scoring

As detailed in Table 3, a hierarchical scoring system was used to assess the presence and severity of diarrhea. Fecal consistency of the calves was evaluated twice daily (07:00 and 20:00 h) by two trained veterinarians following the method of Larson et al. (1977). Consistency was assessed using a 3-point scale, where 0 indicated firm but not hard and 3 indicated watery feces. A fecal consistency score ≥2 was classified as diarrhea (Renaud et al., 2020). Interobserver agreement among veterinarians was evaluated prior to the study using Fleiss’ kappa (0.81). Following the experiment, the average fecal score and diarrhea frequency were calculated using the following formulas:


Average fecal score=Sumof fecal scores/Number of calves;

$$\begin{array}{l} \mathrm{Diarrhea frequency}\;\left(\%\right)=\left(\begin{array}{l} \mathrm{Number of diarrheic calves}\times \\ \mathrm{diarrheic days} \end{array}\right)/\\ \left(\begin{array}{l} \mathrm{Total number of calves}\times \\ \mathrm{Experimental days} \end{array}\right)\times 100. \end{array}$$

**Table 3:** Fecal scoring criteria.

Score	Trait
1	Without fetid odor and hard firm feces
2	Without fetid odor but slightly soft feces
3	With a light fetid odor and soft, partially formed feces
4	With a distinct fetid odor and loose, semiliquid feces
5	With a pungent fetid odor and watery, mucous-like feces

### Blood parameter analysis

Serum samples were thawed at room temperature and gently vortexed before analysis. Biochemical parameters were measured at the Clinical Chemistry Laboratory of the Third People’s Hospital of Xinjiang Uygur Autonomous Region (a tertiary-care facility) using an automated biochemical analyzer (Cobas 8000, Roche Diagnostics, Switzerland). The measured parameters included blood urea nitrogen (BUN), glucose (GLU), alanine aminotransferase (ALT), aspartate aminotransferase (AST), ALT/AST ratio, total bilirubin (TBIL), alkaline phosphatase (ALP), total protein (TP), albumin (ALB), globulin (GLOB), ALB/GLOB ratio, triglycerides (TG), total cholesterol (TC), and creatinine (CRE).

Serum IgG (Kit No. H106-1-1), IgA (Kit No. H108-1-1), IgM (Kit No. H109-1-1), IL-2 (Kit No. H003-1-1), and IFN-γ (Kit No. H025-1-1) and antioxidant indices [superoxide dismutase (SOD, Kit No. A001-3-2), glutathione peroxidase (GSH-Px, Kit No. A005-1-2), total antioxidant capacity (T-AOC, Kit No. A015-2-1), catalase (CAT, Kit No. A007-1-1), and malondialdehyde (MDA, Kit No. A003-1-2)] were determined using commercial kits (Nanjing Jiancheng Bioengineering Institute, Nanjing, China) according to the manufacturer’s instructions.

### Microbiota diversity analysis

Based on growth, immune, antioxidant, and diarrhea data, 500 mg/day per calf was selected as the optimal *B. megaterium* dose for microbiota analysis. Thus, in this study, Groups I and II served as control groups, while Group IV (500 mg/day *B. megaterium*) served as the treatment group. This design enables mechanistic exploration of the optimal-dose phenotype but does not resolve dose–microbiota relationships across all probiotic levels. Rectal content samples were thawed, and DNA was extracted using a kit (Tiangen Biochemical Technology, Beijing, China) under aseptic conditions. DNA concentration was assessed via 0.8% agarose gel electrophoresis, and purity was determined using a multimode microplate reader (Infinite M200, Tecan, Switzerland). To ensure representativeness and avoid selection bias, six samples per group were selected for 16S rRNA gene amplicon sequencing (Beijing Novogene Bioinformatics Co., Ltd.) using a stratified random sampling method based on diarrhea incidence and initial body weight. Qualified DNA served as the template for PCR amplification of the bacterial 16S rRNA gene V3–V4 region, using the barcoded primers 341F (5′-CCTACGGGNGGCWGCAG-3′) and 806R (5′-GGACTACHVGGGTWTCTAAT-3′). PCR products were purified and analyzed by 2% agarose gel electrophoresis in 1× TAE buffer. Target bands were excised, and PCR products were purified and recovered using the GeneJET PCR Purification Kit (Thermo Fisher Scientific). DNA libraries were constructed using the NEBNext Ultra II DNA Library Prep Kit (New England Biolabs, Inc.), quantified with a Qubit fluorometer, and sequenced on the Illumina MiSeq platform (Illumina, Inc., San Diego, CA, United States).

Raw sequences were processed using a 97% similarity operational taxonomic unit (OTU) clustering pipeline. Briefly, primer sequences were trimmed, and paired-end reads were merged using USEARCH v7.0. Quality filtering was applied to remove reads with an expected error threshold >1.0. Chimeric sequences were identified and removed using the *de novo* mode of the UCHIME algorithm as implemented in USEARCH. High-quality, non-chimeric sequences were then clustered into OTUs at a 97% similarity threshold using the UPARSE-OTU algorithm within USEARCH. From each OTU, the most abundant sequence was selected as the representative sequence. Taxonomic assignment of OTU representative sequence was analyzed by RDP Classifier (version 2.2) against the Silva database (version 138.1) using a confidence threshold of 0.7.

Microbiota α-diversity indices (observed OTUs, Shannon, Simpson, and Chao1) were calculated using on rarefied OTU tables. β-diversity was assessed using unweighted UniFrac distances. The statistical significance of group differences in microbial community composition was tested using PERMANOVA with 999 permutations. Results were visualized via principal coordinate analysis (PCoA). Venn diagrams depicting OTU overlap among groups were generated using the Novogene online tool. Centered log-ratio (CLR) transformed genera abundances were correlated with serum indices via Spearman analysis; *p*-values were FDR-corrected (Benjamini–Hochberg, *q* < 0.05). The corrplot package visualized results in R (Wang et al., 2025).

### Statistical analysis

Raw data were managed using Microsoft Excel. Statistical analyses were performed in IBM SPSS Statistics 31.0. Statistical differences among groups were determined by one-way ANOVA followed by Duncan’s multiple range test for *post-hoc* comparisons. This test was selected due to its high sensitivity in detecting potential differences across multiple groups, which aligns with the exploratory aim of this study to identify candidate microbial taxa associated with dietary intervention (Yang et al., 2024; Ma et al., 2025). Differences were considered significant at *p* < 0.05.

## Results

### Growth performance and diarrhea in neonatal calves

The effects of *B. megaterium* supplementation on growth performance are shown in Table 4. No significant difference in body weight (BW) was observed among the groups at the initial stage (1 day, *p* > 0.05). At day 28, BW in Groups II–V was higher than in Group I (*p* < 0.05). However, no significant difference was found between Groups III, IV, V, and Group II (*p* > 0.05). There were no significant differences in DMI between treatment groups at each period. Calves in Group II, IV, and V showed significantly higher ADG from 1 to 14 days compared to Group I (*p* = 0.029). From days 15 to 28, ADG was higher in Groups II–V than in Group I (*p* = 0.041). Over days 1–28, ADG was higher in the supplemented groups than in Group I (*p* = 0.038), with Group II showing the numerically highest value (Table 4). It is noteworthy that Group III, Group IV, and Group V showed no significant differences compared to Group II (*p* > 0.05). Consistent with these gains, F/G was greater in Group I than in the supplemented groups during days 1–14, 15–28, and 1–28 (*p* = 0.048, 0.041, and 0.037, respectively).

**Table 4:** Growth performance of neonatal calves supplemented with *B. megaterium*.

Items	Days	Group I	Group II	Group III	Group IV	Group V	SEM	*p*-value
BW (kg)	1 day	38.33	37.67	38.21	37.95	38.09	6.18	0.881
	14 days	41.38^b^	42.96^a^	42.05^a^	43.02^a^	42.86^a^	6.10	0.473
	28 days	47.80^b^	50.21^a^	49.32^a^	50.14^a^	49.87^a^	2.36	0.038
DMI (g/day)	1–14 days	830.62	802.37	807.12	801.06	799.34	26.93	0.624
	15–28 days	1098.72	1085.44	1090.53	1082.26	1089.66	70.20	0.311
	1–28 days	964.67	947.51	943.47	941.66	958.2	19.13	0.774
ADG (g/day)	1–14 days	218.01^b^	357.86^a^	274.30^ab^	362.33^a^	341.17^a^	92.54	0.029
	15–28 days	458.36^b^	517.79^a^	519.08^a^	508.41^a^	502.92^a^	81.08	0.041
	1–28 days	338.19^b^	417.86^a^	400.76^a^	428.37^a^	421.17^a^	29.27	0.038
F/G	1–14 days	3.81^a^	2.12^b^	2.94^ab^	2.21^b^	2.34^b^	0.19	0.048
	15–28 days	2.40^a^	2.10^b^	2.10^b^	2.19^b^	2.16^b^	0.28	0.041
	1–28 days	2.85^a^	2.12^b^	2.35^b^	2.20^b^	2.28	0.20	0.037

BW, body weight; ADG, average daily gain; DMI, dry matter intake; F/G, ratio of DMI to ADG. ^a,b^Different lowercase letters indicate significant differences (*p* < 0.05). Group I: control group (basal diet); Group II: antibiotic group (basal diet + 50 mg/day per calf gentamicin); Group III: basal diet + 250 mg/day per calf *B. megaterium*; Group IV: basal diet + 500 mg/day per calf *B. megaterium*; Group V: basal diet + 1,000 mg/day per calf *B. megaterium*.

Dietary treatments significantly affected fecal score and diarrhea frequency (Table 5). Average fecal score varied among groups (*p* = 0.026). Calves in Group I had a higher fecal score than those in Groups II, III, IV, and V (*p* < 0.05). Diarrhea frequency also differed among treatments (*p* = 0.045). Groups II, IV and V had lower diarrhea frequency (9.97–12.38%) than Groups I and III (14.96–15.45%; *p* < 0.05).

**Table 5:** Effect of *B. megaterium* on diarrhea in calves.

Items	Group I	Group II	Group III	Group IV	Group V	SEM	*p*-value
Average fecal score	2.50^b^	1.63^a^	2.09^a^	1.69^a^	1.72^a^	0.238	0.026
Diarrhea frequency (%)	15.45^a^	9.97^b^	14.96^a^	10.86^b^	12.38^b^	2.860	0.045

^a,b^Different lowercase letters indicate significant differences (*p* < 0.05). Group I: control group (basal diet); Group II: antibiotic group (basal diet + 50 mg/day per calf gentamicin); Group III: basal diet + 250 mg/day per calf *B. megaterium*; Group IV: basal diet + 500 mg/day per calf *B. megaterium*; Group V: basal diet + 1,000 mg/day per calf *B. megaterium*.

### Serum biochemical parameters

Serum biochemical parameters in calves supplemented with *B. megaterium* are presented in Table 6. Serum GLU concentrations differed significantly among groups (*p* = 0.029). Group II had the lowest GLU level (3.86 mmol/L), which was significantly lower than other groups (*p* < 0.05). ALP also varied significantly (*p* = 0.048), with higher values in Groups I and IV, compared to Groups II, III, and V. ALB concentrations differed significantly among the groups (*p* = 0.025). Group IV had the highest ALB level (35.74 g/L), which was higher than those in other groups, Group I had intermediate levels (33.20 g/L). TG levels showed significant differences (*p* = 0.033), with Group IV higher than Groups I, II, III, and V. No significant differences were observed in other parameters.

**Table 6:** Effect of *B. megaterium* on serum biochemical parameters in calves.

Items	Group I	Group II	Group III	Group IV	Group V	SEM	*p*-value
BUN (mmol/L)	5.23	6.84	4.10	3.55	4.52	0.91	0.624
GLU (mmol/L)	4.28^b^	3.86^c^	4.84^b^	5.15^a^	4.32^b^	0.69	0.029
ALT (U/L)	28.47	32.56	29.21	26.83	27.24	9.21	0.081
AST (U/L)	85.40	111.62	35.21	34.85	34.20	9.94	0.066
TBIL (μmol/L)	5.84	7.24	6.54	6.26	5.925	1.68	0.511
ALP (U/L)	199.77^a^	199.38^b^	188.55^b^	202.32^a^	192.12^b^	55.91	0.048
TP (g/L)	63.55	61.54	60.60	65.67	61.52	11.98	0.052
ALB (g/L)	31.54^b^	31.20^b^	30.46^b^	35.74^a^	33.20^ab^	11.27	0.025
GLOB (g/L)	29.54	30.27	30.42	29.70	30.45	60.76	0.056
ALB/GLOB	1.13	1.04	0.97	1.18	1.04	0.48	0.349
TG (mmol/L)	0.55^b^	0.52^b^	0.50^b^	0.62^a^	0.52^b^	0.35	0.033
TC (mmol/L)	3.35	3.15	3.20	3.51	3.15	1.20	0.931
CRE (μmol/L)	62.74	60.90	59.22	65.24	61.72	14.58	0.065

^a–c^Different lowercase letters indicate significant differences (*p* < 0.05). Group I: control group (basal diet); Group II: antibiotic group (basal diet + 50 mg/day per calf gentamicin); Group III: basal diet + 250 mg/day per calf *B. megaterium*; Group IV: basal diet + 500 mg/day per calf *B. megaterium*; Group V: basal diet + 1,000 mg/day per calf *B. megaterium*.

### Serum immune parameters

Serum immunoglobulin and cytokine concentrations in calves supplemented with *B. megaterium* are presented in Table 7. IgG concentrations differed significantly among the groups (*p* = 0.024). Group I had the lowest level, significantly lower than other groups (*p* < 0.05). IL-1β concentration was lowest in Group IV, significantly lower than in other groups (*p* = 0.038). IL-10 levels showed significant differences (*p* = 0.045), with the lowest concentrations in Group III, which were lower (*p* < 0.05) than those in Groups I, II, IV, and V. TNF-α concentrations differed among groups (*p* = 0.036), with Group IV exhibiting the lowest levels, lower than those in Groups I, II, III, and V (*p* < 0.05). Similarly, IFN-γ levels varied significantly (*p* = 0.023), with higher concentrations in Group I compared to other groups.

**Table 7:** Effect of *B. megaterium* on serum immune parameters in calves.

Items	Group I	Group II	Group III	Group IV	Group V	SEM	*p*-value
IgG (g/L)	6.54^c^	8.20^b^	8.25^b^	9.74^a^	7.75^bc^	0.22	0.024
IgA (g/L)	0.84	0.61	0.82	1.01	0.74	0.29	0.124
IgM (g/L)	0.43	0.31	0.84	0.50	0.38	0.26	0.076
IL-1β (ng/L)	12.36^a^	15.95^a^	14.85^a^	9.95^b^	13.45^a^	3.85	0.038
IL-2 (ng/L)	12.13	8.45	17.78	15.25	10.94	0.97	0.068
IL-6 (ng/L)	14.78	11.44	17.81	12.02	16.35	4.88	0.085
IL-10 (pg/L)	18.30^b^	21.63^b^	15.35^c^	25.96^a^	26.52^a^	6.31	0.045
TNF-α (ng/L)	14.93^a^	16.81^a^	18.38^a^	11.92^b^	16.36^a^	4.80	0.036
IFN-γ (ng/L)	48.38a	35.15^b^	21.71^c^	23.41^c^	30.15^bc^	9.01	0.023

^a–c^Different lowercase letters indicate significant differences (*p* < 0.05). Group I: control group (basal diet); Group II: antibiotic group (basal diet + 50 mg/day per calf gentamicin); Group III: basal diet + 250 mg/day per calf *B. megaterium*; Group IV: basal diet + 500 mg/day per calf *B. megaterium*; Group V: basal diet + 1,000 mg/day per calf *B. megaterium*.

### Serum antioxidant parameters

As shown in Table 8, GSH-Px activity differed among groups and was higher in Groups IV and V than in Groups I–III (*p* = 0.025). CAT activity also differed among groups (*p* = 0.038). Group I had the lowest CAT activity, Groups II and III had intermediate values, and Groups IV and V had the highest values. MDA concentrations showed significant differences (*p* = 0.032), with lower levels in Groups III, IV, and V, compared to Groups I and II. SOD and T-AOC did not differ significantly among groups.

**Table 8:** Effect of *B. megaterium* on serum antioxidant indicators in calves.

Items	Group I	Group II	Group III	Group IV	Group V	SEM	*p*-value
SOD (U/mL)	40.48	46.29	52.05	58.12	62.85	13.35	0.651
GSH-Px (U/mL)	246.82^b^	256.03^b^	286.28^b^	310.56^a^	315.72^a^	86.84	0.025
CAT (U/mL)	18.99^c^	23.18^b^	27.75^ab^	32.46^a^	32.89^a^	7.52	0.038
T-AOC (U/mL)	4.92	5.31	5.63	5.91	6.22	0.91	0.843
MDA (nmol/mL)	7.14^a^	6.65^a^	6.18^b^	5.82^b^	5.85^b^	1.13	0.032

^a–c^Different lowercase letters indicate significant differences (*p* < 0.05). Group I: control group (basal diet); Group II: antibiotic group (basal diet + 50 mg/day per calf gentamicin); Group III: basal diet + 250 mg/day per calf *B. megaterium*; Group IV: basal diet + 500 mg/day per calf *B. megaterium*; Group V: basal diet + 1,000 mg/day per calf *B. megaterium*.

### Bacterial community diversity

**Figure 1:**
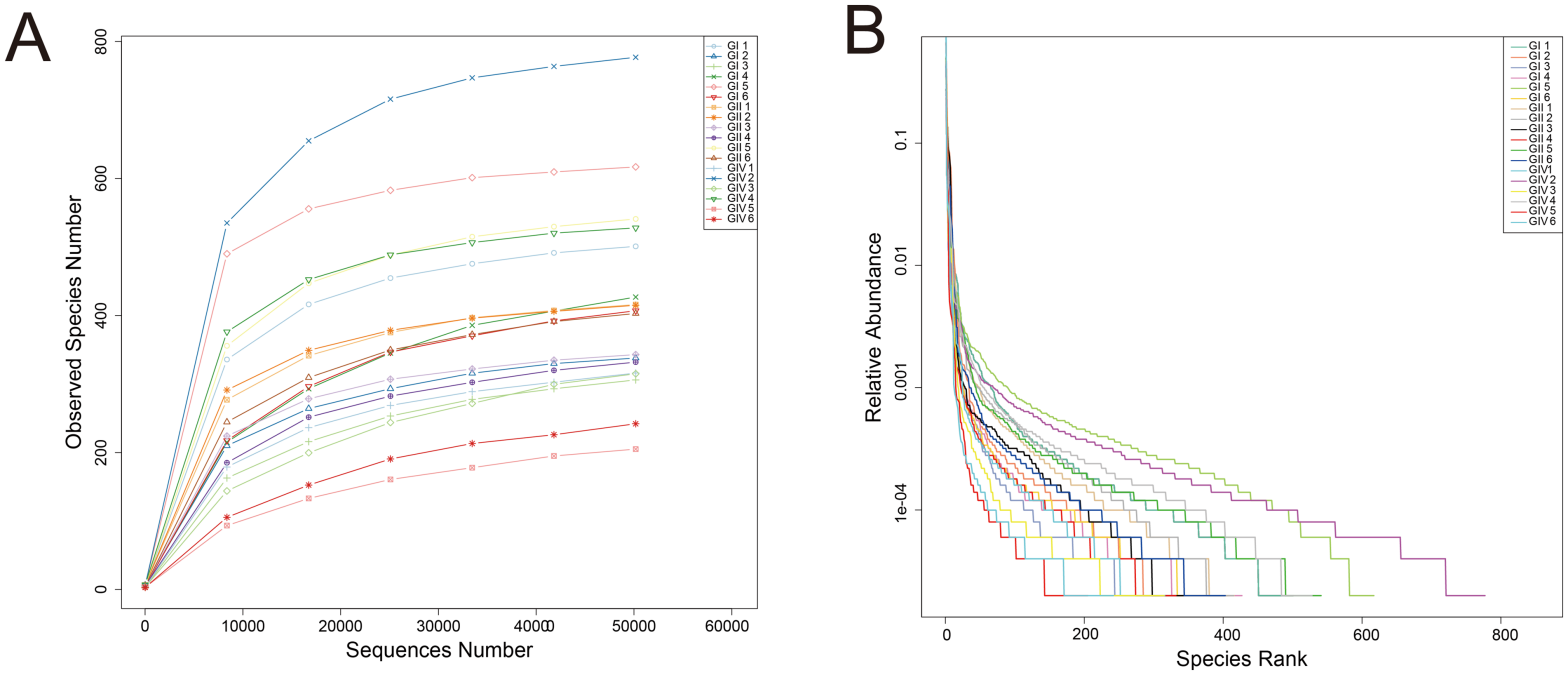
Quality assessment of 16S rRNA gene amplicon sequencing for rectal contents in calves. **(A)** Rarefaction curves of rectal microbial communities. **(B)** Rank-abundance curves of rectal microbial communities.

The validity of 16S rRNA Gene Amplicon Sequencing was assessed using sample rarefaction (Figure 1A) and rank-abundance curves (Figure 1B). The plateau observed at the end of the rarefaction curve confirmed adequate sequencing depth, while the progressive flattening of the rank-abundance curve with increasing sequencing depth validated the species richness measurements.

**Figure 2:**
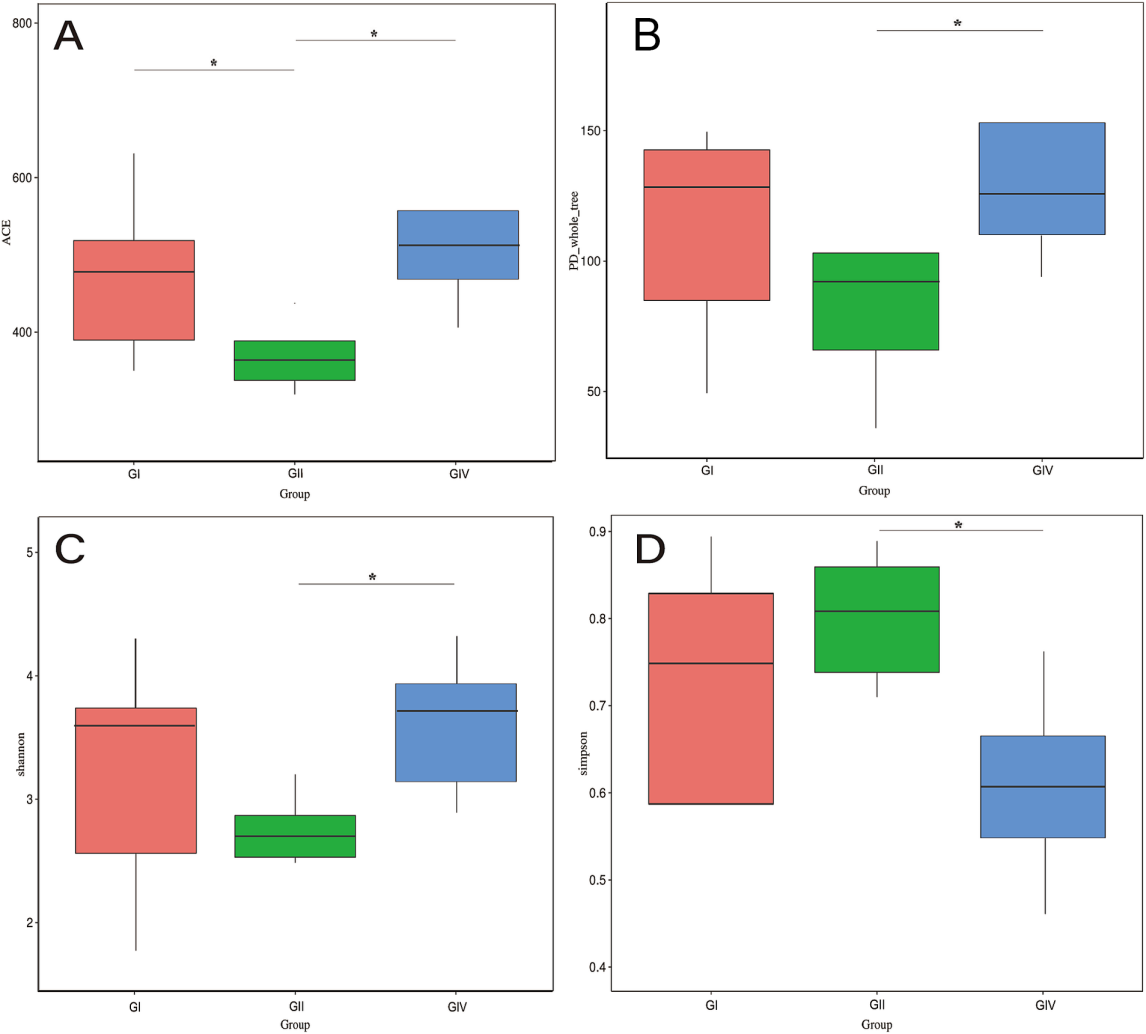
α-diversity analysis of rectal microorganisms in newborn calves following *B. megaterium* supplementation. **(A)** ACE index, **(B)** Chao 1 index, **(C)** Shannon index, and **(D)** Simpson index box plots compare microbial richness and diversity across GI, GII, and GIV groups. Asterisks (*) indicate statistically significant differences between groups, highlighting the influence of *B. megaterium* on rectal microbial community structure. GI: control group (basal diet); GII: antibiotic group (basal diet + 50 mg/day per calf gentamicin); GIV: *B. megaterium* group (basal diet + 500 mg/day per calf *B. megaterium*).

As shown in Figure 2A, the ACE index indicated that microbial richness was significantly highest in the GIV group, intermediate in the GI group, and lowest in the GII group, with significant differences between GIV and GII, as well as between GI and GII (*p* < 0.05). The Chao1 index (Figure 2B) showed a similar trend; the difference between GIV and GII was statistically significant (*p* < 0.05). The Shannon index (Figure 2C) was significantly greater in the GIV group, compared to the GII group (*p* < 0.05). Conversely, the Simpson index (Figure 2D) demonstrated significantly lower diversity in the GII group compared with the GIV group (*p* < 0.05).

**Figure 3:**
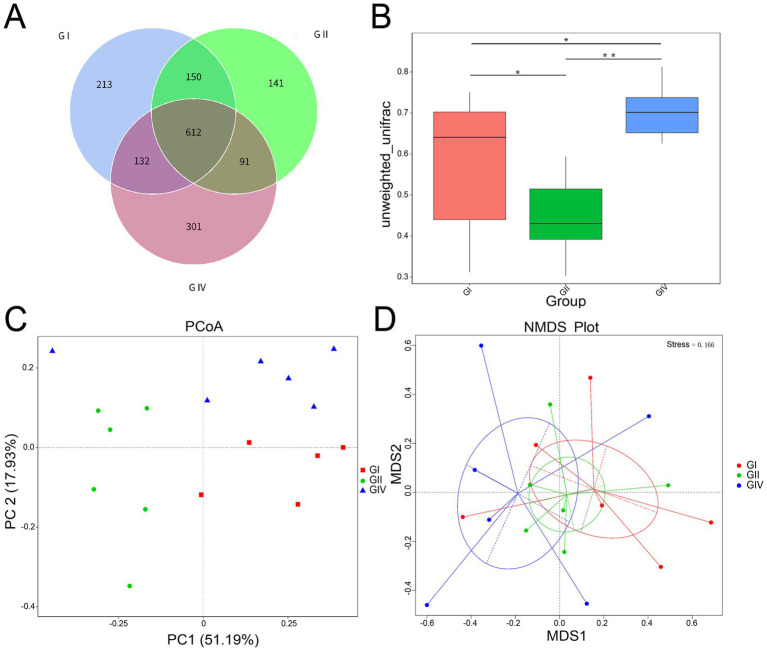
β-diversity analysis of rectal microorganisms in calves following *B. megaterium* supplementation. **(A)** Venn diagram of shared and unique operational taxonomic units (OTUs). **(B)** Box plot of beta diversity (unweighted unifrac distance). **(C)** PCoA, **(D)** NMDS. GI: control group (basal diet); GII: antibiotic group (basal diet + 50 mg/day per calf gentamicin); GIV: *B. megaterium* group (basal diet + 500 mg/day per calf *B. megaterium*).

As illustrated in Figure 3A, a total of 612 OTUs were shared by Groups I, II, and IV. In addition, 213, 141, and 301 OTUs were unique to Groups I, II, and IV, respectively. Unweighted UniFrac distances (Figure 3B) indicated marked differences in microbial community structure among groups (ANOSIM: *R* = 0.3209, *p* = 0.004). Notably, Group IV exhibited a distinct clustering pattern compared to Groups I and II. Principal coordinate analysis (PCoA, Figure 3C) and non-metric multidimensional scaling (NMDS, Figure 3D) further separated samples by treatment. Samples from the same group tended to cluster closely, while clear separations among groups, particularly between Group IV and the others, were observed.

### Bacterial community composition

**Figure 4:**
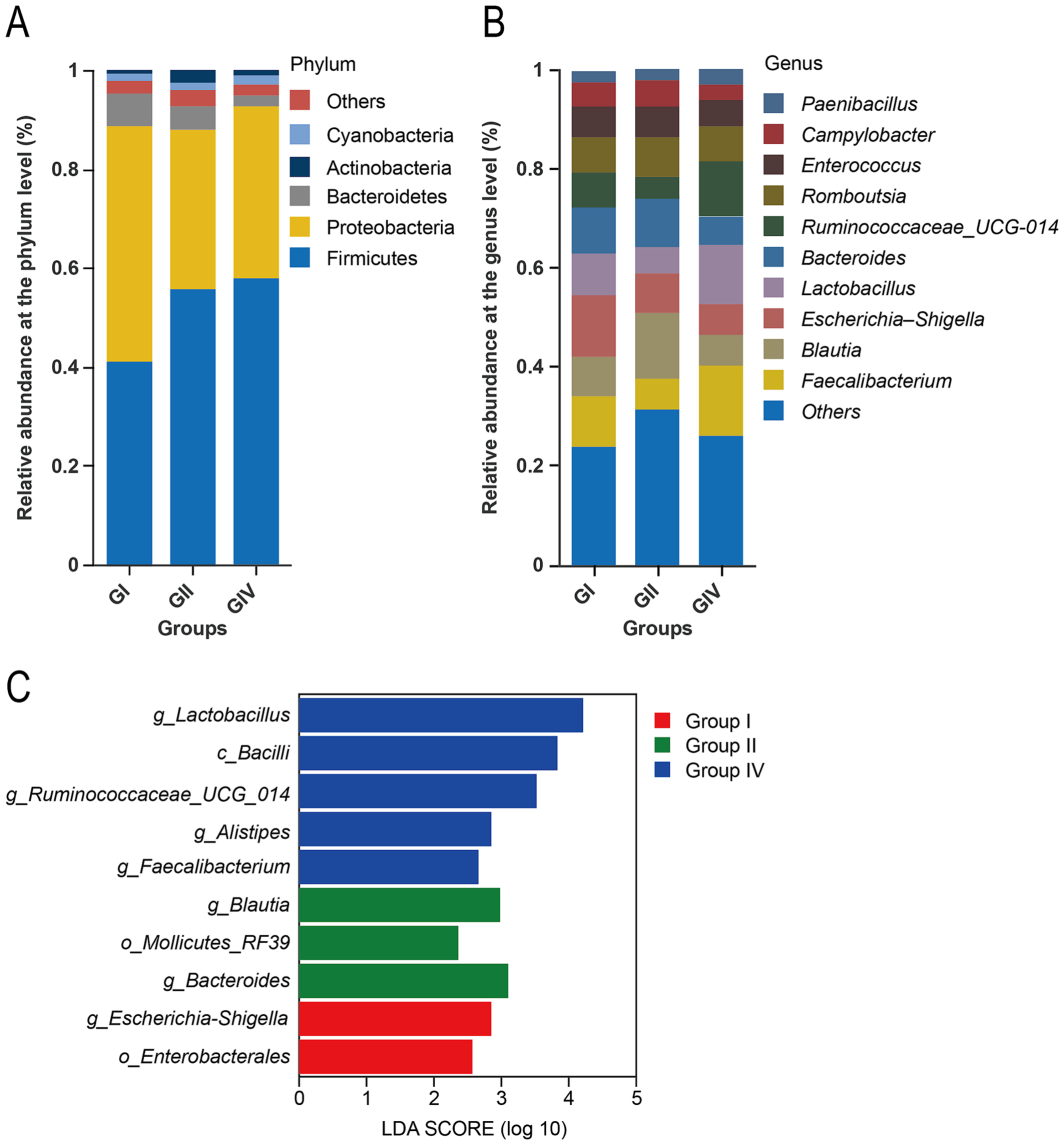
Effects of *B. megaterium* supplementation on rectal microbiota composition in neonatal calves. **(A)** Bacterial community composition at the phylum level. **(B)** Bacterial community composition at the genus level. **(C)** Linear discriminant analysis (LDA) effect size (LEfSe) showing significantly enriched taxa among groups. Group I: control group (basal diet); Group II: antibiotic group (basal diet + 50 mg/day per calf gentamicin); Group IV: *B. megaterium* group (basal diet + 500 mg/day per calf *B. megaterium*).

Figures 4A,B show the five most abundant phyla and the 10 most abundant genera. At the phylum level, Firmicutes were the predominant group, followed by Proteobacteria, Bacteroidetes, Cyanobacteria, and Actinobacteria. The relative abundance of Firmicutes significantly increased from Group I to Group IV (*p* = 0.015), while Proteobacteria and Bacteroidetes declined progressively (*p* < 0.05).

At the genus level, *Faecalibacterium* (6.03–14.3%) and *Bacteroides* (5.31–10.02%) were the predominant genus in the rectal contents, followed by *Lactobacillus* (5.21–15.1%), and *Escherichia–Shigella* (3.21–12.3%). After intervention, the abundance of *Lactobacillus* and *Faecalibacterium* significantly increased in Group IV compared to Group I (*p* = 0.009 and *p* = 0.012, respectively), while *Escherichia–Shigella* showed a significant reduction (*p* = 0.018). Similarly, *Ruminococcaceae_UCG-014* was significantly enriched in Group IV (*p* = 0.011), whereas *Bacteroides* and Blautia exhibited group-dependent variations (*p* = 0.035 and *p* = 0.027, respectively). No significant differences were found in the relative abundance of Romboutsia, Enterococcus, Campylobacter, or Paenibacillus (*p* > 0.05).

Linear discriminant analysis effect size (LEfSe) was performed to identify taxa with differential abundance among groups, using a logarithmic LDA score and non-parametric factorial Kruskal–Wallis test (LDA >2.0, *p* < 0.05) on the relative abundance data. LEfSe identified differential taxa among groups (Figure 4C). Group IV was characterized by a significant enrichment of *Lactobacillus*, *Ruminococcaceae_UCG_014*, and *Faecalibacterium*. In contrast, Group II demonstrated marked enrichment of *Bacteroides* and *Blautia*. Additionally, Group I was significantly associated with *Escherichia–Shigella*. These preliminary differential microbial signatures, identified via LEfSe highlight compositional differences between groups and suggest candidate taxa that may be involved in the host response to *B. megaterium* supplementation.

### Correlation analysis between differential apparent indices and microorganisms

**Figure 5:**
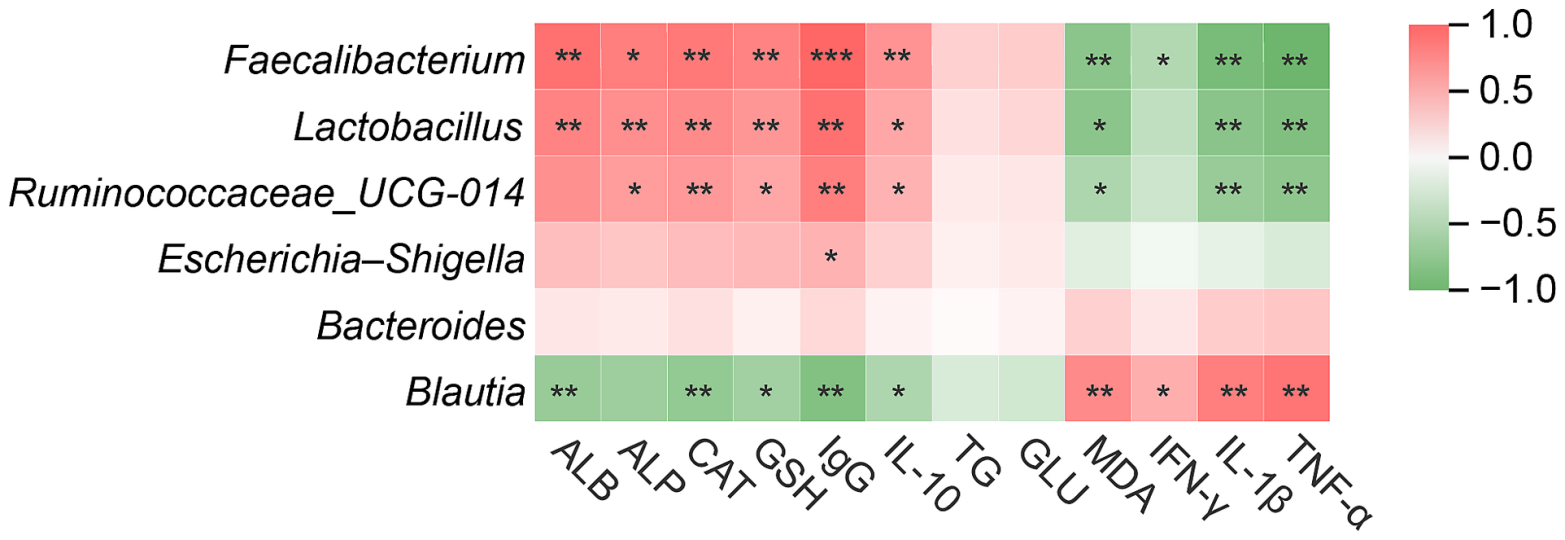
Correlation analysis between differential serum indices and key microbial taxa in calves. Red and green indicate positive and negative correlations, respectively, and color intensity reflects correlation strength. Asterisks indicate significant differences (^*^p < 0.05, ^**^p < 0.01, and ^***^p < 0.001).

A correlation heatmap was generated to examine associations between differential serum indices and key microbial taxa (Figure 5). *Faecalibacterium* displayed strong positive correlations with ALB, ALP, CAT, GSH-Px, IgG, IL-10, and TG, whereas it was negatively associated with GLU, IFN-γ, IL-1β, and TNF-α. *Lactobacillus* exhibited significant positive correlations with antioxidant and immune-related parameters (CAT, GSH-Px, IgG, IL-10) and negative correlations with GLU. *Ruminococcaceae_UCG-014* was positively correlated with ALB, ALP, CAT, GSH-Px, IgG, IL-10, and TG, and negatively correlated with GLU and IFN-γ. In contrast, *Escherichia–Shigella* showing negative correlations with antioxidant and immune indicators (CAT, GSH-Px, IgG, IL-10) and positive correlations with GLU, MDA, IFN-γ, IL-1β, and TNF-α. *Bacteroides* displayed weak but significant negative correlations with IgG and IL-10. Blautia was positively correlated with ALB and ALP, while showing negative associations with IL-1β and TNF-α.

## Discussion

This study evaluated dietary *B. megaterium* supplementation on growth, diarrhea, serum biochemical, immune antioxidant indices, and rectal microbiota in neonatal calves exposed to cold conditions in Xinjiang, China. The medium dose (500 mg/day per calf) improved growth, reduced diarrhea, enhanced humoral immunity and antioxidant status, and shifted rectal microbiota towards a more beneficial profile. These combined physiological and microbial changes indicate that *B. megaterium* may be a useful alternative to antibiotics for improving calf health in cold regions.

### Effect of *Bacillus megaterium* on growth performance and diarrhea incidence in calves

In calves receiving *B. megaterium*, ADG increased and F/G decreased compared with the basal diet, indicating a growth-promoting effect. Diarrhea frequency was also lower in the probiotic group, and in some cases comparable to or lower than the antibiotic group. Similar improvements in ADG and feed use with *B. megaterium* have been reported in Holstein bull calves (Yao et al., 2020; Deng et al., 2021). The reduction in diarrhea with *B. megaterium* is important because it suggests a potential to support both growth performance and gastrointestinal health, which could help reduce reliance on in-feed antibiotics and associated concerns such as antimicrobial resistance (Vanbelle et al., 1990). The growth response may be related to enzyme production (e.g., amylases, proteases) by *B. megaterium*, which can improve nutrient digestion and absorption (Rai et al., 2017; Cai et al., 2019). The reduced diarrhea may be associated with immunomodulation and inhibition of pathogenic bacteria. Enrichment of genera such as *Lactobacillus* and reduction of *Escherichia–Shigella* are consistent with improved microbial balance and may contribute to lower diarrhea risk (Li et al., 2023; Kong et al., 2024). The observation that the antibiotic group showed high ADG but less pronounced improvement in diarrhea than the probiotic group highlights distinct modes of action. These findings position *B. megaterium* as a promising candidate for further evaluation as part of strategies aimed at optimizing calf health and growth while minimizing antibiotic use. Future studies incorporating non-inferiority trials and farm-scale economic and health outcome assessments are warranted to substantiate its practical application.

### Effect of *Bacillus megaterium* on serum biochemical parameters in calves

In this study, 500 mg/day *B. megaterium* increased GLU, ALB, and TG concentrations in neonatal calves. All measured values remained within the established normal physiological ranges for healthy neonatal calves (Yu D. et al., 2019; Yu K. et al., 2019). Higher GLU may reflect improved energy supply, which is important for growth and thermoregulation under cold stress (Sun G. et al., 2022; Sun Q. et al., 2022). Besides, GLU impacts immune homeostasis under chronic cold stress, which can be crucial for overall health and growth (Sun G. et al., 2022; Sun Q. et al., 2022). Increased ALB and the tendency for higher TP may indicate better protein status, which supports tissue growth and immune function (Yu D. et al., 2019; Yu K. et al., 2019; Che et al., 2024). These findings align with studies in piglets showing increased TP and ALB with *B. megaterium* supplementation (Deng et al., 2021). Higher TG levels may be related to changes in lipid metabolism, including fat absorption or hepatic lipid handling (Ye et al., 2020). Unlike some studies that report stable or decreased TG, this divergence might be attributed to the specific physiological demands of neonatal calves, breed differences, or the unique environmental stressors of Xinjiang. Under cold stress, neonatal calves have high thermogenic requirements (Zhao et al., 2018). The elevated serum GLU and TG observed in the probiotic group suggest an improved capacity to mobilize energy substrates to maintain body temperature and homeostasis, preventing the hypoglycemia often seen in energy-deficient, cold-stressed neonates. However, it is acknowledged that sustained or excessive increases in TG could be a marker of metabolic dysregulation in other contexts. Similar ALT and AST values between control and *B. megaterium* groups, together with higher AST in the antibiotic group, suggest that the probiotic did not adversely affect liver function and may be safer than antibiotics in this respect (Florek et al., 2023). This is particularly important for neonatal calves, whose immature metabolic systems can be easily overwhelmed. The proposed mechanisms include enhanced nutrient breakdown by bacterial enzymes, reduced intestinal inflammation leading to decreased endotoxin translocation, and direct modulation of host metabolic pathways by microbial metabolites (Liang et al., 2020; Wu et al., 2022).

### Effect of *Bacillus megaterium* on serum immune parameters in calves

*B. megaterium* supplementation was associated with higher immunoglobulin concentrations and lower pro-inflammatory cytokines in neonatal calves. The 500 mg/day dose increased serum IgG compared with the basal diet. Pro-inflammatory cytokines (IL-1β, TNF-α and IFN-γ) decreased, whereas the anti-inflammatory cytokine IL-10 increased in Groups IV and V. These findings are consistent with previous research demonstrating *B. megaterium* to enhance humoral immunity and mitigate inflammatory responses in chickens and piglets (Bakun et al., 2021; Luise et al., 2022). The elevation of IgG levels is critical for neonatal calves, whose passive immunity from colostrum wanes rapidly, leaving them vulnerable to pathogens (Quigley et al., 2001). The concomitant reduction in pro-inflammatory cytokines and the induction of IL-10 suggest a shift towards an anti-inflammatory state, which is beneficial for maintaining gut integrity and overall immune homeostasis under stress (Neurath, 2024). These immunomodulatory effects may involve interactions between bacterial cell wall components (e.g., peptidoglycan) and host pattern recognition receptors such as TLR2 (Wolf and Underhill, 2018; Wang et al., 2019; Sun Q. et al., 2022). Furthermore, the modulation of gut microbiota by *B. megaterium* could potentially enhance host immunity. We hypothesize that this may occur through the production of microbial metabolites such as short-chain fatty acids (SCFAs), which are known to influence immune cell development and function (Liu et al., 2023). Future studies directly measuring SCFA concentrations and gut permeability markers would be valuable to test this hypothesis.

### Effect of *Bacillus megaterium* on serum antioxidant capacity in calves

Neonatal calves under environmental stressors, are highly susceptible to oxidative stress due to their immature antioxidant systems (Zhang et al., 2024). *B. megaterium* supplementation, particularly at 500 mg/day, increased GSH-Px and CAT activities and reduced MDA, indicating improved antioxidant status. While superoxide dismutase SOD and T-AOC showed upward trends, they did not reach statistical significance. These results align with studies in aquatic animals (Monier et al., 2023; Elbahnaswy et al., 2024) and broilers (Luise et al., 2022), where *Bacillus* spp. (including *B. megaterium*) improved antioxidant enzyme activities and reduced lipid peroxidation; Furthermore, *B. megaterium* has demonstrated a direct effect in reducing MDA and alleviating oxidative stress in both calves and mammalian models (Mazzoli et al., 2019; Yao et al., 2020). Notably, the antioxidant benefits conferred by *B. megaterium* were superior to those observed in the antibiotic group. Several mechanisms may underlie the antioxidant effects of *B. megaterium*. Certain *Bacillus* strains are known to produce their own antioxidant enzymes (e.g., Mn-SOD), which could contribute to scavenging reactive oxygen species (Ji et al., 2025). *B. megaterium* can enrich beneficial gut bacteria (e.g., *Lactobacillus*) that produce antioxidative metabolites such as SCFAs and phenolic compounds (Feng and Wang, 2020). Additionally, cell wall components of *B. megaterium* may activate host antioxidant pathways, such as the Nrf2 pathway, which upregulates the expression of endogenous antioxidant enzymes like SOD and GSH-Px (Wolf and Underhill, 2018; Wang et al., 2019; Sun G. et al., 2022; Sun Q. et al., 2022). By bolstering the antioxidant defense, *B. megaterium* helps mitigate cellular damage caused by free radicals, reduces oxidative stress, and contributes to the health and resilience of calves facing harsh environmental conditions (Yao et al., 2020). However, the functions of *B. megaterium* are inferred from the literature rather than directly measured. The present data support the host-microbiota associations but do not validate specific metabolic pathways. Therefore, future studies integrating ranscriptomics of the Nrf2 pathway, quantification of bacterial antioxidant enzyme expression, or metabolomic profiling of recal contents are needed to confirm the functional mechanisms.

### Effect of *Bacillus megaterium* on rectal microbiota composition in calves

*B. megaterium* supplementation altered rectal microbiota composition. In Group IV, α-diversity were higher in controls, suggesting increased richness and diversity (Li et al., 2023). Notably, *B. megaterium* supplementation significantly increases the relative abundance of Firmicutes while decreasing that of Proteobacteria and Bacteroidetes. In neonatal calves, these shifts warrant careful interpretation. An increase in the Firmicutes-to-Bacteroidetes (F/B) ratio has been frequently observed in association with improved energy harvest in some models (Li et al., 2023), though its functional implications in the rapidly developing neonatal gut are complex and not fully defined. The reduction in Proteobacteria may be indicative of a less inflammatory gut environment (Shin et al., 2015), which aligns with the observed reduction in diarrhea and pro-inflammatory cytokines. The decrease in Bacteroidetes suggests a broader reorganization of the microbial community structure under the influence of *B. megaterium*. At the genus level revealed that *B. megaterium* supplementation significantly enriched beneficial genera such as *Lactobacillus*, *Faecalibacterium*, and *Ruminococcaceae_UCG-014* in Group IV. *Lactobacillus* are well-known probiotics that produce lactic acid, bacteriocins, and other antimicrobial compounds, inhibiting pathogen growth and strengthening the intestinal barrier (Kong et al., 2024). *Faecalibacterium*, particularly *Faecalibacterium prausnitzii*, is a prominent butyrate producer, which is a crucial energy source for colonocytes and possesses potent anti-inflammatory properties (Li et al., 2023). *Ruminococcaceae_UCG-014* belongs to a family of bacteria known for their role in fiber digestion and SCFA production, contributing to gut health and energy metabolism (Deng et al., 2021). In contrast, the relative abundance of *Escherichia–Shigella*, which includes important enteric pathogens, was lower in the *B. megaterium* group. This direct suppression of pathogens, coupled with the promotion of beneficial bacteria, is a critical mechanism by which *B. megaterium* mitigates diarrhea and improves gut health. The LEfSe analysis further corroborated these findings, identifying *Lactobacillus*, *Ruminococcaceae_UCG-014*, and *Faecalibacterium* as key biomarkers for the *B. megaterium* group, while *Escherichia–Shigella* was a significant biomarker for the control group. These compositional changes are consistent with a shift towards a more stable microbial community and may contribute to the observed health benefits. However, although gentamicin achieved comparable growth performance to the *B. megaterium*, the probiotic and antibiotic groups differed in diarrhea outcomes and microbiota patterns. In 16S profiles, the gentamicin group showed distinct community and lower diversity relative to the 500 mg/day *B. megaterium* group, whereas *B. megaterium* enriched *Lactobacillus* and *Faecalibacterium*, reduced *Escherichia–Shigella*. Given concerns about antimicrobial resistance and microbiota disruption, *B. megaterium* may serve as a complementary strategy emphasizing host resilience rather than antibiotic-like effects.

### Correlation analysis between serum parameters and rectal microorganisms

Correlations between key genera and serum indices provide clues about links between microbiota and host physiology in neonatal calves. *Faecalibacterium*, *Lactobacillus*, and *Ruminococcaceae_UCG-014* showed positive associations with ALB, ALP, CAT, GSH-Px, IgG, and IL-10 and negative associations with GLU, IFN-γ, IL-1β, and TNF-α. *Faecalibacterium* and *Ruminococcaceae_UCG-014*, established butyrate and SCFA producers, enhance gut barrier integrity and reduce inflammation (Flint et al., 2012; Louis et al., 2014). Their associations with ALB and TG further indicate improved protein synthesis and lipid metabolism essential for calf growth (Deng et al., 2021). Enhanced antioxidant enzymes and immunoglobulins, together with reduced pro-inflammatory cytokines, reflect a stable, anti-inflammatory metabolic state, consistent with SCFA-mediated immune benefits (Morrison and Preston, 2016; Riaz Rajoka et al., 2021). It is important to note that the relationships between rectal microbiota and serum indices observed here are correlational. While the data suggests a link between microbial shifts and improved health, further work using fecal transplantation or gnotobiotic models is necessary to establish causality. *Lactobacillus* showed similar positive correlations with antioxidant and immune markers, reinforcing its known immunomodulatory roles (Gareau et al., 2010; Valeriano et al., 2017). Blautia also correlated positively with ALB and ALP and negatively with IL-1β and TNF-α, supporting its reported anti-inflammatory potential (Liu et al., 2021).

In contrast, *Escherichia–Shigella*, identified as a predominant genus in the control group, displayed negative correlations with critical antioxidant and immune indicators (CAT, GSH-Px, IgG, IL-10) and strong positive correlations with GLU, MDA, IFN-γ, IL-1β, and TNF-α. This pattern strongly implicates *Escherichia–Shigella* in driving oxidative stress, inflammation, and metabolic dysregulation, which are hallmarks of gastrointestinal disturbances and diarrhea in calves (Chen et al., 2022; Zhang L. et al., 2025; Zhang Z. et al., 2025). The elevation of MDA, a marker of lipid peroxidation, alongside increased pro-inflammatory cytokines, underscores the detrimental impact of these opportunistic pathogens on host health. The weak negative correlations of *Bacteroides* with IgG and IL-10 might suggest that, in the context of dysbiosis in neonatal calves, certain *Bacteroides* species could contribute to a less optimal immune state, although the role of *Bacteroides* is highly context-dependent and species-specific (Scher et al., 2013). Overall, *B. megaterium* intervention effectively suppressed *Escherichia–Shigella* and promoted the beneficial genera, thereby shifting these correlations towards a healthier physiological state.

Although this study focuses on cold environments, neonatal calves are subjected to various stressors (such as heat stress, weaning, transportation, and pathogen challenges), which can also lead to oxidative stress, immune dysregulation, barrier damage, and microbiota imbalance. In this study, the coordinated improvement of immune, antioxidant indicators, and bacterial composition in calves supplemented with *B. megaterium* suggests that *B. megaterium* may also exert a positive effect on these negative factors. However, this needs to be validated in further experiments. Future studies could extend the experimental period for calves to investigate the sustainability of *B. megaterium* on immunity, antioxidants, and microbiota changes, and integrate metabolomics or metagenomics to link taxonomic shifts with underlying mechanistic pathways.

## Conclusion

Supplementing neonatal calves with *B. megaterium*, particularly at 500 mg/day, yielded growth performance comparable to the antibiotic group, while also reducing diarrhea, enhanced serum immune and antioxidant status, and increasing the beneficial genera relative abundance of *Lactobacillus*, *Faecalibacterium*, and *Ruminococcaceae_UCG-014* while decreasing *Escherichia–Shigella*. The observed shifts in the gut microbiota are consistent with concurrent improvements in host physiological indices, suggesting that *B. megaterium* may aid calves in coping with cold stress. The study provides evidence that *B. megaterium* could be considered as a potential alternative to antibiotics in calf rearing. However, these interpretations should be considered in light of limitations, including the subset analysis of microbiota (*n* = 6 per group), the need for a consistently reported bioinformatics pipeline, and the lack of direct functional readouts linking microbial changes to host phenotypes. Future work should clarify microbial functional pathways and evaluate long-term health and production outcomes of early-life *B. megaterium* supplementation.

## Data Availability

The data presented in this study are publicly available. The data can be found here: https://www.ncbi.nlm.nih.gov, accession PRJNA1438074.
